# Multicenter Observational Retrospective Study on Febrile Events in Patients with Acute Myeloid Leukemia Treated with Cpx-351 in “Real-Life”: The SEIFEM Experience

**DOI:** 10.3390/cancers15133457

**Published:** 2023-07-01

**Authors:** Luana Fianchi, Fabio Guolo, Francesco Marchesi, Chiara Cattaneo, Michele Gottardi, Francesco Restuccia, Anna Candoni, Elettra Ortu La Barbera, Rita Fazzi, Crescenza Pasciolla, Olimpia Finizio, Nicola Fracchiolla, Mario Delia, Federica Lessi, Michelina Dargenio, Valentina Bonuomo, Maria Ilaria Del Principe, Patrizia Zappasodi, Marco Picardi, Claudia Basilico, Monica Piedimonte, Paola Minetto, Antonio Giordano, Patrizia Chiusolo, Lucia Prezioso, Caterina Buquicchio, Lorella Maria Antonia Melillo, Daniele Zama, Francesca Farina, Valentina Mancini, Irene Terrenato, Michela Rondoni, Irene Urbino, Mario Tumbarello, Alessandro Busca, Livio Pagano

**Affiliations:** 1 Dipartimento di Diagnostica per Immagini, Radioterapia Oncologica ed Ematologia, Fondazione Policlinico Universitario A. Gemelli, IRCCS-Università Cattolica del Sacro Cuore, 00168 Roma, Italy; antonio.giordano@guest.policlinicogemelli.it (A.G.); patrizia.chiusolo@unicatt.it (P.C.); livio.pagano@unicatt.it (L.P.); 2 IRCCS Ospedale Policlinico San Martino, 16132 Genova, Italy; fabio.guolo@hsanmartino.it; 3 IRCCS Istituto Nazionale Tumori Regina Elena, 00128 Rome, Italy; francesco.marchesi@ifo.it; 4 SC Ematologia e Dipartimento di Oncologia Clinica, A.O. Spedali Civili, 25123 Brescia, Italy; chiara.cattaneo@asst-spedalicivili.it; 5 Onco Hematology, Department of Oncology, Veneto Institute of Oncology, IOV-IRCCS, 31033 Padua, Italy; michele.gottardi@iov.veneto.it; 6 Ospedale Civile, 65124 Pescara, Italy; francesco.restuccia@live.com; 7 Dipartimento di Scienze Mediche e Chirurgiche Materno-Infantili e dell’Adulto, Università degli Studi di Modena e Reggio Emilia, 41125 Modena, Italy; anna.candoni@unimore.it; 8 UOC Ematologia con Trapianto Santa Maria Goretti, 04100 Latina, Italy; e.ortulabarbera@hotmail.it; 9 Hematology Unit—A.O.U.P. Ospedale Santa Chiara, 56126 Pisa, Italy; r.fazzi@hotmail.it; 10 Haematology Unit, IRCCS Istituto Tumori “Giovanni Paolo II”, 70124 Bari, Italy; crescenza.pasciolla@gmail.com; 11 AORN Cardarelli, 80131 Napoli, Italy; olimpiafinizio@gmail.com; 12 Fondazione IRCCS Cà Granda-Ospedale Maggiore Policlinico di Milano, 20122 Milano, Italy; nicola.fracchiolla@policlinico.mi.it; 13 Hematology and Transplant Unit, Azienda Ospedaliero-Universitaria Consorziale Policlinico di Bari, 70124 Bari, Italy; mario.delia74@gmail.com; 14 Ematologia e Immunologia, Clinica Azienda Ospedaliera di Padova, 35128 Padova, Italy; federica.lessi@unipd.it; 15 UO Ematologia e Trapianto CSE V. Fazzi, 73100 Lecce, Italy; miviforina@tiscali.it; 16 UOC Ematologia, Policlinico Borgo Roma, 37134 Verona, Italy; 17 Dipartimento di Biomedicina e Prevenzione, Università degli studi di Roma “Tor Vergata”, 00133 Roma, Italy; del.principe@med.uniroma2.it; 18 Fondazione IRCCS Policlinico San Matteo, 27100 Pavia, Italy; p.zappasodi@smatteo.pv.it; 19 Ematologia, AOU Federico II, 80131 Napoli, Italy; 20 Azienda Socio Sanitaria Territoriale dei Sette Laghi, 21100 Varese, Italy; claudiamaria.basilico@asst-settelaghi.it; 21 AOU Sant’Andrea, 00189 Roma, Italy; monica.piedimonte@uniroma1.it; 22 Ematologia e Trapianto, IRCCS Ospedale Policlinico San Martino, 16132 Genova, Italy; paola.minetto@hsanmartino.it; 23 Ematologia e CTMO, AOU Parma, 43126 Parma, Italy; lprezioso@ao.pr.it; 24 Sc Ematologia Con Trapianto, Ospedale Dimiccoli, 70051 Barletta, Italy; caterinabuquicchio@libero.it; 25 Ematologia, Ospedale Casa Sllievo della Sofferenza, 71013 San Giovanni Rotondo, Italy; lmelillo@ospedaliriunitifoggia.it; 26 Policlinico Sant’Orsola Malpighi, 40138 Bologna, Italy; daniele.zama@gmail.com; 27 U.O. Ematologia e Trapianto Midollo, Dipartimento di Oncologia, Istituto Scientifico San Raffaele, 20132 Milano, Italy; farina.francesca@hsr.it; 28 Divisione di Ematologia, Ospedale Niguarda Milano, 20162 Milano, Italy; valentina_mancini_mi@hotmail.com; 29 UOSD Clinical Trial Center e Biostatistica e Bioinformatica, IRCCS Istituto Nazionale Tumori Regina Elena, 00128 Roma, Italy; irene.terrenato@ifo.it; 30 U.O.C. di Ematologia, Azienda Unità Sanitaria Locale della Romagna, 48124 Ravenna, Italy; michela.rondoni@auslromagna.it; 31 SC Ematologia, Ospedale AOU Città Della Salute e della Scienza, 10126 Torino, Italyabusca@cittadellasalute.to.it (A.B.); 32 Department of Medical Biotechnologies, University of Siena, 53100 Siena, Italy; mario.tumbarello@unisi.it

**Keywords:** secondary acute myeloid leukemia, CPX-351 therapy, febrile events

## Abstract

**Simple Summary:**

CPX-351 has been approved for the treatment of adults with therapy-related AML (t-AML) or AML with myelodysplasia-related changes (AML–MRC). The aim of this retrospective study was to evaluate the absolute infectious risk in a real-life setting of 200 AML patients treated with this drug. A total of 249 febrile events were recorded in 336 courses of CPX. The attributable mortality–infection rate in our series was 6%, confirming a good safety profile for CPX-351, with an incidence of infectious complications comparable to that of the pivotal studies. The only factor that was significantly associated with mortality in the multivariate analysis was the lack of response to CPX-351 treatment.

**Abstract:**

In the present study, we aimed to evaluate the absolute risk of infection in the real-life setting of AML patients treated with CPX-351. The study included all patients with AML from 30 Italian hematology centers of the SEIFEM group who received CPX-351 from July 2018 to June 2021. There were 200 patients included. Overall, 336 CPX-351 courses were counted: all 200 patients received the first induction cycle, 18 patients (5%) received a second CPX-351 induction, while 86 patients (26%) proceeded with the first CPX-351 consolidation cycle, and 32 patients (10%) received a second CPX-351 consolidation. A total of 249 febrile events were recorded: 193 during the first or second induction, and 56 after the first or second consolidation. After the diagnostic work-up, 92 events (37%) were classified as febrile neutropenia of unknown origin (FUO), 118 (47%) were classifiable as microbiologically documented infections, and 39 (17%) were classifiable as clinically documented infections. The overall 30-day mortality rate was 14% (28/200). The attributable mortality–infection rate was 6% (15/249). A lack of response to the CPX-351 treatment was the only factor significantly associated with mortality in the multivariate analysis [*p*-value: 0.004, OR 0.05, 95% CI 0.01–0.39]. Our study confirms the good safety profile of CPX-351 in a real-life setting, with an incidence of infectious complications comparable to that of the pivotal studies; despite prolonged neutropenia, the incidence of fungal infections was low, as was infection-related mortality.

## 1. Introduction

CPX-351 is a liposomal formulation of cytarabine and daunorubicin in a 5-to-1 molar ratio approved for treatment of adult patients with therapy-related acute myeloid leukemia (t-AML) and AML with myelodysplasia-related changes (AML–MRC) [1,2]. In vitro studies demonstrated that this formulation maximizes the synergy between cytarabine and daunorubicin, and markedly increases the half-life of both agents, contributing to their effective penetration and accumulation in the bone marrow [3,4]. This pharmacokinetic characteristic, however, results in an increased time to treatment response and prolonged post-chemotherapy cytopenia, with a consequent potential increase in risk of infections. In previous studies, recovery from neutropenia occurred around 36 days after initiation of CPX-351 (vs. 32 days with traditional chemotherapy), with a variable incidence in infections and febrile neutropenia [1,2,5,6,7,8,9,10].

In the present study, we aimed to evaluate the absolute infectious risk in a real-life setting of AML patients treated with CPX-351. The secondary endpoints evaluated in the study were type, incidence, and outcome of bacterial, fungal, and viral infections and infection-related mortality rate, with stratification by disease subtype.

## 2. Materials and Methods

This retrospective multicenter study included all consecutive patients with AML from 30 Italian hematology centers of the SEIFEM group (Sorveglianza Epidemiologica Infezioni nelle Emopatie) who received at least 1 course of CPX-351 from July 2018 to June 2021. The study was approved by the ethics committee of the coordinating center, Fondazione Policlinico Universitario Agostino Gemelli—IRCCS, Università Cattolica del Sacro Cuore of Rome, Italy (Study ID: 3405), and by the respective ethics committees of all participating centers; written informed consent for data collection was obtained from each patient enrolled. The study was conducted according to the Declaration of Helsinki.

The main enrollment criteria were diagnosis of secondary t-AML or AML–MRC according to the WHO 2016 definitions, and treatment with at least 1 CPX-351 course. Forms were sent to all centers for data collection. Specifically, for each course of chemotherapy performed, a data collection form was compiled, including information on the underlying disease, response to treatment (complete or partial remission, refractory), any prophylaxis performed, other potential risk factors for infection (e.g., use of steroids, previous exposure to hypomethylating agents, previous infectious events, and severity and duration of neutropenia). With regard to infections, detailed information was collected including the onset, time, signs and symptoms, site, etiology, treatment, and course. All of the collected data were encoded in a specific database and analyzed.

Overall, a complete set of data was collected for 200 patients with AML who had received CPX-351 in the 30 participating hematology centers.

The standard schedule for CPX-351 treatment was used and consisted of the following:

Induction course with a CPX-351 dose of 44 mg/m^2^ (daunorubicin 44 mg/m^2^ plus cytarabine 100 mg/m^2^) repeated on days 1, 3, and 5; a second induction with the same dose of the drug given on days 1 and 3 for patients failing to achieve at least CRi after the first induction cycle; consolidation courses with a CPX-351 dose of 29 mg/m^2^ (daunorubicin 29 mg/m^2^ plus cytarabine 65 mg/m^2^) repeated on days 1 and 3.

Patients were withdrawn from the observational study at the time of transplantation.

### Statistical Analysis

All of the variables of interest were summarized through descriptive statistics. We used frequencies and percentage values for categorical variables, while continuous variables were reported with median values and their relative ranges. Comparisons between groups were tested by the Pearson’s chi-square non-parametric test. Overall survival (OS) analyses were carried out using Cox proportional hazard regression models. The hazard risks (HR) and their relative 95% confidence intervals (95% CI) were estimated for each variable, adopting the most suitable prognostic modality as the reference group. Multivariate models were conducted considering the variables that were significant in the univariate analysis using the forward selection method. In order to better understand the clinical role of CPX-351 we decided to consider the response to this treatment as a potential factor in the survival model, which is a widespread methodological approach in clinical studies. A *p*-value < 0.05 was considered statistically significant. The statistical analysis was performed using SPSS software (SPSS version 21, SPSS Inc., Chicago, IL, USA).

## 3. Results

### 3.1. Treatment and Response

There were 200 patients with AML (103 male/97 female) with a median age of 65 y (range 18–80, 141 aged over 60) that were included in this study: 78 with AML–MRC (38%), 69 (35%) with AML secondary to a previous myelodysplastic syndrome (sAML), and 53 (26%) with t-AML (Table 1). Forty patients (20%) had received prior treatment with hypomethylating agents for a previous diagnosis of myelodysplastic syndrome.

Overall, in the study period, 336 courses of CPX-351 were administered: all 200 patients received the first induction cycle; 18 patients (5%) received a second CPX-351 induction, while 86 patients (26%) proceeded with CPX-351 consolidation; 32 patients (10%) received a second consolidation cycle.

The majority of patients (184/200, 94%) received antifungal prophylaxis with posaconazole during CPX-351 treatment, while an antibacterial with quinolone or antiviral prophylaxis with aciclovir was administered in 109 (56%) and 92 (47%) patients, respectively. A history of other infections prior to CPX-351 treatment was reported in 23 patients (11.5%). Interestingly, 14 of these 23 were patients with AML post-MDS who had experienced the reported infection during the myelodysplastic phase; in the other 9 patients, the reported infection occurred at the time of AML diagnosis.

All of the patients experienced severe neutropenia (PMN < 0.1  ×  10^9^/L) following CPX-351 treatment, and the median time to neutrophil recovery (>0.5  ×  10^9^/L) was 30 days (range 13–80) after the first or second induction, and 17 days (range 10–40) after consolidation. G-CSF treatment was performed, as febrile neutropenia prophylaxis in both induction and consolidation, in 85 of the 336 CPX-351 courses (25%) for a median time of 14 days (range 1–45).

The overall response rate (ORR) to CPX-351 treatment was 69.5%: CR/CRi in 122/200 patients (61.5%), PR in 18/200 (9.5%), no response in 49/200 (24.5%), and not evaluable in 11/200 (5.5%) due to early death before evaluation.

Eighty-eight of the included patients (44%) underwent allogeneic bone marrow transplantation post-CPX-351 treatment by the time of closure of the observational study period.

### 3.2. Febrile Events

A total of 249 febrile events per 336 courses of CPX-351 (74%) were recorded, with a significant difference in incidences between induction/reinduction and consolidation, with 193 febrile events occurring during the first or second induction (89%), and 56 events occurring after the first and second consolidation (47%) (*p* < 0.0001). In Table 2, the characteristics of all 249 febrile events are shown, divided by treatment phase (induction vs. reinduction vs. consolidation).

Overall, after a complete diagnostic work-up, 39 febrile events (16%) were classifiable as clinically documented infections, 118 (47%) were classifiable as microbiologically documented infections, while 92 (37%) were classified as febrile neutropenia of unknown origin (FUO).

The clinically documented infections included pneumonia (*n* = 24), cellulitis/abscesses (*n* = 11), arthritis (*n* = 3), mucositis (*n* = 1), and sinusitis (*n* = 1).

**Figure 1:**
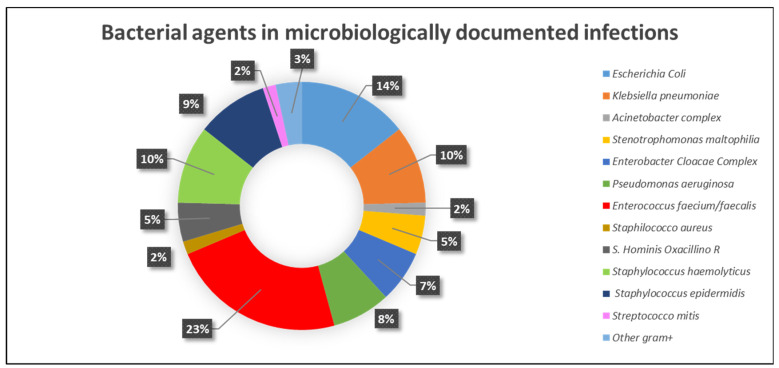
Bacterial agents in microbiologically documented infections.

The characteristics of microbiologically documented infections are reported in Table 3. Most of the microbiologically documented infections were of bacterial origin (105/118), while fungal and viral infections occurred in 11 and 2 cases, respectively. Characterization of the bacterial agents is reported in Figure 1.

In particular, bacteremia occurred in 102 cases (30%): 84 (82%) during induction/reinduction, and 18 (18%) during consolidation. In 9/102 cases (9%), concomitant pneumonia was present. Other microbiologically documented infections included pneumonia (*n* = 6), abscesses (*n* = 2), cystitis (*n* = 5), discitis (*n* = 1), and sinusitis (*n* = 2), with orbital involvement in 1 case. Bacteremia was due to Gram-positive bacteria in 58 cases (57%), Gram-negative bacteria in 36 cases (35%), and mixed in 8 cases (8%).

Overall, fungal infections were diagnosed in 11 cases (5.5%) (including 1 case associated with bacterial sepsis), and was classifiable as proven in 3 cases (2 Aspergillus spp. and 1 Pneumocystis jirovecii pneumonia), probable aspergillosis in 7 cases, and possible aspergillosis in 1 case. The sites of aspergillosis were lung in 9 cases and orbital in 1 case. Interestingly, all of the fungal infections occurred during the induction phase. Viral infection was reported in 2 cases: 1 case of SARS-CoV-2 pneumonia and 1 case of CMV reactivation associated with bacteremia. Antibacterial treatment was performed in all 249 febrile events, while in 69 cases, antifungal therapy was also given.

### 3.3. Mortality

The overall 30-day mortality rate was 14% (28/200); the majority of deaths (22/28) occurred during a first (*n* = 19; 9.5%) or second induction cycle (*n* = 3/18; 16%), while 6 deaths occurred during a consolidation cycle (5%). The cause of death was infection in 15 patients, AML progression in 8 patients, and cerebral hemorrhage and cardiac complications in 3 and 2 patients, respectively. The mortality attributable to the febrile event rate was 6% (15/249; 8 bacterial origin, 1 fungal origin, 1 SARS-CoV-2 pneumonia, 4 cases of clinically documented pneumonia, and 1 FUO). Specifically, 12/15 (80%) of the deaths were attributed to infection occurring during the induction/reinduction cycles, and 3/15 (20%) during consolidation cycles. Notably, all infection-related deaths occurred in patients who were refractory to CPX-351 treatment. The median overall survival was 17.9 months (range 0.6–39.6+).

### 3.4. Risk Factors for Mortality

Data on the risk of infection-related mortality at 30 days are shown in Table 4. Factors that were significantly associated with 30-day infection-related mortality in the induction cycle in univariate analysis were previous infection [*p*-value 0.006, HR 3.84, 95% CI 1.46–10.10)] and lack of response to CPX-351 treatment [*p*-value 0.003, HR 0.04, 95% CI 0.01–0.33). A lack of response to CPX-351 treatment was the only factor that remained statistically significant in the multivariate analysis [*p*-value 0.004, HR 0.05, 95% CI 0.01–0.39)]. Neither the type of event (clinically documented vs. microbiologically documented vs. FUO) nor the type of microbiologically documented infection (bacterial vs. fungal) significantly influenced early mortality at 30 days.

## 4. Discussion

Infectious complications following treatment of AML represent an important cause of morbidity and mortality, with risk of infection even higher in patients with myelodysplastic features resulting in impairments in neutrophil bactericidal and fungicidal activity [11]. The risk of infectious complications is highly variable, and is influenced by patient characteristics including age, comorbidities, degree of immunodeficiency, and disease status, as well as by the use of infection prophylaxis and the chemotherapy protocol. The “3 + 7” regimen “(Cytarabine + Daunorubicin)”, which represents the gold standard for the treatment of AML, contributes to a heightened infection risk in AML patients as a result of the toxicity induced at the level of the gut mucous membranes [12]. A study by Hueso et al. investigated the impact of induction chemotherapy on the intestinal barrier both in AML patients and in a murine model. In the AML patients, they demonstrated a profound compromise of the intestinal barrier, with transient epithelial damage resulting in a prolonged loss of load, diversity, and function of the microbiota. Using the murine model, the authors more deeply investigated the specific effects of chemotherapy on the gastrointestinal tract, which they characterized as a qualitative dysbiosis and physical barrier impairment that facilitates bacterial translocation [12]. It is hypothesized that CPX-351, as a result of its particular formulation, may have a reduced impact than the “7 + 3” combination on the integrity of the intestinal mucosa. Results from experimental models of intestinal toxicity have shown that, at a variance with the “7 + 3” combination, CPX351 exhibited a barrier protective effect and prevented unwanted inflammation by preserving beneficial microbial composition and function in the gut [13].

The safety profile of CPX-351 appears to be similar to that of 7 + 3, albeit with a more prolonged myelosuppression and a slower recovery from neutropenia and thrombocytopenia as a result of the pharmacokinetics properties of the liposomal formulation, impacting the delivery of daunorubicin and cytarabine to the bone marrow. Despite the more prolonged neutropenia, however, infections occurred with a similar incidence in patients receiving either 7 + 3 or CPX-351 in the pivotal studies (around 92–93%), though patients in the CPX-351 arm did experience a higher rate of serious infections (32% vs. 21%), particularly of bacteremia. Importantly, however, this difference did not translate into a higher rate of discontinuation of the study treatment or overall AE-related mortality [1,2,9].

As shown in Table 5, we compared the incidence of infectious events in the present study with those of previous studies on CPX-351. Compared to previously published studies that reported an incidence of febrile neutropenia as high as 91% of cases during CPX-351 treatment [5,7,8], in our study, we observed a lower incidence, which was comparable to that reported in the pivotal studies (74%) [1,2]. Not all previously published studies observed an increased incidence of febrile neutropenia in induction with CPX-351 compared with 3 + 7. In the study by Lancet et al., febrile neutropenia was reported in 68% of patients treated with CPX-351 and in 71% of patients who received the standard 3 + 7 chemotherapy [1].

In an expanded access program, 52 patients were treated with CPX-351 for 1–2 induction cycles and up to 4 consolidation cycles. The most common serious adverse events reported during treatment with CPX-351 were febrile neutropenia (19%), pneumonia (10%), and infection (8%), with 30- and 60-day mortality rates of 0% and 6%, respectively [6]. In a cohort of 71 patients treated with CPX-351 according to the Italian Compassionate Use Program (CUP), infections were most common adverse event, with FUO occurring in 20 patients (28%), sepsis in 20 patients (28%), pneumonia in 8 patients (11.3%), including two cases of *Pneumocystis jirovecii*-related pneumonia (PCP), and invasive fungal infections in 3 patients (4.2%). The 60-day treatment-related mortality due to uncontrolled infections, however, was low (4.2%) [7]. Rautenberg et al. analyzed data from 188 consecutive patients who received CPX-351 induction chemotherapy as first-line therapy for AML: in 2-years of follow-up, infectious complications were the most frequent adverse events (AE) of grade 3 and higher among non-hematologic toxicities (22%), with febrile neutropenia reported in 15% and pneumonia in 22% of patients; gastrointestinal side effects, including mucositis, were reported in only 4% of cases. The 30-day early death rate was 8% overall, and was significantly higher in patients 65 years and above [10]. The incidence of serious infections in the present study was low, which is reflected in the observed low rate of infection-related mortality, and refractoriness to CPX-351 was the only factor significant for 30-day infection-related mortality. Sepsis occurred in 30% of cases and accounted for 42% of all febrile events, with the majority of events associated with Gram-positive bacteria. For comparison, the incidence of infections in a similar population of patients with therapy-related myeloid neoplasms treated with less intensive chemotherapy (azacytidine alone) was 16%, with an infection-related mortality of 4% [14]. In a recent study by Matthews et al. comparing the real-world effectiveness of CPX-351 vs. venetoclax and azacytidine, the incidence of infections was higher in patients who received CPX-351 (51% vs. 20%), though mortality rates were similar between the two groups (5%) [15].

In the present study, CPX-351 was associated with a low incidence of fungal infections. All of the reported fungal infections occurred during induction, which is consistent with previous studies that showed that the highest incidence of invasive fungal infections occurred during the first remission induction cycle [16,17,18]. Probable/proven and possible invasive fungal diseases (IFD) were observed in *n* = 10 (5%) and *n* = 1 (2%) patients, respectively, most of whom received posaconazole as antifungal prophylaxis. For comparison, Cattaneo et al. reported an incidence of probable/proven and possible invasive fungal diseases (IFD) of 10.5% and 9.7%, respectively, in 114 patients with FLT-3 mutated AML treated with 3 + 7 + midostaurin. In the 48% of patients in their study population who received posaconazole only as antifungal prophylaxis, the incidence of proven/probable IFD was 9.1% (16). In our study, the incidence of fungal infections (5.5%) was lower than those reported in previous studies with data on patients treated with CPX-351; in those studies, fungal infections were more frequent in patients treated with CPX-351 compared with 3 + 7 (14% vs. 2.4%; 10% vs. 1%) [1,5].

The aspergillosis-attributable mortality rate (AMR) in AML is generally around 30–40%. In two consecutive multicenter studies, it was observed that the AMR decreased from 48% in 1987–1998 to 38.5% in 1999–2003 [19]. In our series, the aspergillosis-attributable mortality rate was 0.4% (1/249 events). The low incidence of aspergillosis in our study is a very important finding, not only in regard to mortality but also because it has been demonstrated that invasive aspergillosis during induction therapy can delay the subsequent therapeutic program and have a significant impact on OS, particularly in AML patients who fail to attain a CR with the first cycle of induction therapy [20].

**Table 5:** Incidence of infectious events in the main studies on CPX-351 treatment.

Reference	Study	Patients (*n*°)	Febrile Neutropenia/FUO	Pneumonia	Bacterial Infections	Fungal Infections
Cortes et al., Cancer 2014 [3]	CPX 351	81	44 (54%)	18 ** (22%)	Sepsis: 24 (30%) UTI: 5 (6%)	See Pneumonia
	Control arm	44	14 (34%)	4 (9%)	Sepsis: 19 (43%) UTI: 5 (11%)	
Lancet et al., Blood 2014 [4]	CPX 351	85	54 (63%)	13 (15%)	30 (35%)	12 (14%) IFI
	3 + 7	41	21 (51%)	8 (19%)	8 (20%)	1 (2.4%) IFI
Lancet et al., J Clin Oncol 2018 [1]	CPX 351	153	68%	20%	nr	nr
	3 + 7	156	71%	15%	nr	nr
Issa et al. Leukemia 2020 [8]	Phase 2	56	19 (34%)	15 (27%)	Sepsis: 10 (18%) GI: 1 (2%)	nr
Guolo et al., Blood Cancer J 2020 [7]	Phase 4	71	20 (28%)	8 (11%)	20 (28%)	2 *PjP* 3 (4%) IA
Roboz et al., Leuk and Lymph 2020 [6]	Phase 4	52	40 (77%)	7 (13%)	3 (6%)	nr
Chiche et al., Blood Adv 2021 [5]	Phase 4	103	94 (91%)	30 (30%)	25 (24%)	10 (10%) IA 1 (1%) CDC
Rautenberg et al., Blood Cancer J. 2021 [10]	Phase 4	188	28 (15%)	42 (22%)	41 (22%) *	
Matthews AH et al., Blood Adv 2022 [20]	CPX 351	52	47 (90%)	nr	35 (67%)	nr
	Ven/aza	59	32 (54%)	nr	21 (36%)	nr
Present study	Phase 4	200 (336 courses)	92 (37%) ^§^	30 (9%) ^§^	114 (34%) ^§^	11 (5.5%) ^§^: • 10 IA (2 proven, 7 probable, 1 possible) • 1 PJP

* Type of infection not specified; ** includes pneumocystis, bacterial pneumonia, and fungal pneumonia. ^§^ Percentage reported for 336 cycles. Legend: nr: not reported; IA: invasive aspergillosis, PjP: pneumocystis jirovecii pneumonia.

## 5. Conclusions

Albeit with the limitations of a retrospective series, our study confirms the good safety profile of CPX-351 in a real-life setting, with a low infection-related mortality, even in categories of patients considered particularly at risk, including those with myelodysplastic changes. In our experience, CPX-351 was well tolerated by patients, and was associated with a low incidence of fungal infections; importantly, this allowed us to proceed with the therapeutic regimen in all patients who achieved a hematologic response, with 44% of patients going on to receive bone marrow transplantation by the end of the observational study period.

## Figures and Tables

**Table 1:** Characteristics of all enrolled patients.

	N (%)
***n*° of unique patients**	200
**Age at diagnosis**	
• Median (min-max)	65 (18–80)
**Gender**	
• Male	103 (51.5%)
• Female	97 (48.5%)
**Diagnosis**	
• s-AML	69 (34.5%)
• t-AML	53 (26.5%)
• AML–MRC	78 (39%)
**Previous hypomethylating agents**	40 (20%)
**Previous steroid treatment**	31 (15.5%)
**Anti-fungal prophylaxis**	184 (92%)
• Posaconazole	179
• Other	5
**Anti-bacterial prophylaxis**	
• Quinolone	109 (54.5%)
**Anti-viral prophylaxis**	92 (46%)
**Phase of treatment**	
Induction	200
Re-induction	18
1° consolidation	86
2° consolidation	32

**Legend:** s-AML: AML secondary to a previous myelodysplastic syndrome; t-AML: therapy-related acute myeloid leukemia; AML–MRC: AML with myelodysplasia-related changes.

**Table 2:** Febrile events in induction vs. consolidation.

	Induction 1 (*n* = 200) *n* (%)	Consolidation 1+2 (*n* = 118) *n*(%)	*p*-Value
No febrile event	23 (11.5%)	62 (53%)	<0.0001
Febrile Event	177 (88.5%)	56 (47%)	
FUO	62 (31%)	25 (45%)	
microbiologically documented	88 (44%)	21 (37.5%)	
clinically documented	29(13.5%)	10 (18%)	

**Table 3:** Characteristics of clinically and microbiologically documented infections by site and treatment phase.

	Overall	Induction	Re-Induction	1° Consolidation	2° Consolidation
	N	N	N	N	N
Bacteremia	102	76	9	14	3
Micr. doc	102	76	9	14	3
Gram+	58	44	5	7	2
Gram−	36	26	7	3	0
Mixed	8	6	1	0	1
Pneumonia	30	26		3	1
Clin. doc * including: Probable Aspergillosis Possible Aspergillosis	24 7 1	21 7 1	-	2	1
Micr. doc	6	5	-	1	-
Gram+	1	1	-	-	-
Gram−	3	3	-	-	-
Fungal agent ^§^	1	1	-	-	-
Viral ^@^	1	-	-	1	-
Cellulitis/abscesses	13	8	2	2	
Clin doc	11	7	2	2	1
Micr. doc	2	2	-	-	-
Gram+	1	1	-	-	-
Gram−	1	1	-	-	-
Sinusitis	3				
Clin doc	1	-	-	1	-
Micr. doc	2	2	-	-	-
Gram+	1	1	-	-	-
Fungal **	1	1	-	-	-
Cystitis	5	2		2	1
Micr. doc	5	2		2	1
Gram+	1	-	-	-	1
Gram−	4	2	-	2	-

LEGEND: Clin doc: clinically documented, Micr doc: microbiologically documented; * Including 7 probable and 1 possible aspergillosis; ^§^ Pneumocystis jirovecii pneumonia, ^@^ SARS-CoV-2; ** Aspergillus Fumigatus.

**Table 4:** Infection specific survival. Cox models at 1° induction.

		Univariate	
	Comparison	HR (95%CI)	*p*-Value
**Age**	Continuous	1.01 (0.96–1.08)	0.643
**Gender**	M vs. F	0.82 (0.35–1.93)	0.645
**Diagnosis**	t-AML vs. s-AML	0.61 (0.21–1.79)	0.370
	AML–MRC vs. s-AML	0.51 (0.19–1.41)	0.194
**Previous steroids use**	Yes vs. No	1.43 (0.48–4.28)	0.523
**Previuos infection**	Yes vs. No	**3.84 (1.46–10.10)**	**0.006**
**Quinolones prophylaxis**	Yes vs. No	0.48 (0.20–1.16)	0.102
No response to CPX-351 treatment *	CR vs. no CR	**0.04 (0.01–0.33)**	**0.003**
**Type of infections diagnosis**	FUO vs. Microbiologically-clinically documented	0.46 (0.16–1.38)	0.168
**Type of specific infection**	Fungal vs. bacterial	0.05 (0–1275.67)	0.553

* Only the no response to CPX-351 treatment maintained its statistical significance in multivariate analysis [*p*-value 0.004, HR (95% CI) 0.05 (0.01–0.39)].

## Data Availability

The data presented in this study are available in this article.

## References

[1] Lancet J.E., Uy G.L., Cortes J.E., Newell L.F., Lin T.L., Ritchie E.K., Stuart R.K., Strickland S.A., Hogge D., Solomon S.R. (2018). CPX-351 (cytarabine and daunorubicin) Liposome for Injection Versus Conventional Cytarabine Plus Daunorubicin in Older Patients with Newly Diagnosed Secondary Acute Myeloid Leukemia. J. Clin. Oncol..

[2] Lancet J.E., Uy G.L., Newell L.F., Lin T.L., Ritchie E.K., Stuart R.K., Strickland S.A., Hogge D., Solomon S.R., Bixby D.L. (2021). CPX-351 versus 7+3 cytarabine and daunorubicin chemotherapy in older adults with newly diagnosed high-risk or secondary acute myeloid leukaemia: 5-year results of a randomised, open-label, multicentre, phase 3 trial. Lancet Haematol..

[3] Cortes J.E., Goldberg S.L., Feldman E.J., Rizzeri D.A., Hogge D.E., Larson M., Pigneux A., Recher C., Schiller G., Warzocha K. (2014). Phase II, multicenter, randomized trial of CPX-351 (cytarabine:daunorubicin) liposome injection versus intensive salvage therapy in adults with first relapse AML. Cancer.

[4] Lancet J.E., Cortes J.E., Hogge D.E., Tallman M.S., Kovacsovics T.J., Damon L.E., Komrokji R., Solomon S.R., Kolitz J.E., Cooper M. (2014). Phase 2 trial of CPX-351, a fixed 5:1 molar ratio of cytarabine/daunorubicin, vs cytarabine/daunorubicin in older adults with untreated AML. Blood.

[5] Chiche E., Rahmé R., Bertoli S., Dumas P.Y., Micol J.B., Hicheri Y., Pasquier F., Peterlin P., Chevallier P., Thomas X. (2021). Real-life experience with CPX-351 and impact on the outcome of high-risk AML patients: A multicentric French cohort. Blood Adv..

[6] Roboz G.J., Larson M.L., Rubenstein S.E., Solomon S.R., Schiller G.J., An Q., Chiarella M., Louie A.C., Lin T.L. (2020). Final safety and efficacy results from the CPX-351 early access program for older patients with high-risk or secondary acute myeloid leukemia. Leuk. Lymphoma.

[7] Guolo F., Fianchi L., Minetto P., Clavio M., Gottardi M., Galimberti S., Rizzuto G., Rondoni M., Bertani G., Dargenio M. (2020). CPX-351 treatment in secondary acute myeloblastic leukemia is effective and improves the feasibility of allogeneic stem cell transplantation: Results of the Italian compassionate use program. Blood Cancer J..

[8] Issa G.C., Kantarjian H.M., Xiao L., Ning J., Alvarado Y., Borthakur G., Daver N., DiNardo C.D., Jabbour E., Bose P. (2020). Phase II trial of CPX-351 in patients with acute myeloid leukemia at high risk for induction mortality. Leukemia.

[9] Tzogani K., Penttilä K., Lapveteläinen T., Hemmings R., Koenig J., Freire J., Márcia S., Cole S., Coppola P., Flores B. (2020). EMA Review of Daunorubicin and Cytarabine Encapsulated in Liposomes (Vyxeos, CPX-351) for the Treatment of Adults with Newly Diagnosed, Therapy-Related Acute Myeloid Leukemia or Acute Myeloid Leukemia with Myelodysplasia-Related Changes. Oncologist.

[10] Rautenberg C., Stölzel F., Röllig C., Stelljes M., Gaidzik V., Lauseker M., Kriege O., Verbeek M., Unglaub J.M., Thol F. (2021). Real-world experience of CPX-351 as first-line treatment for patients with acute myeloid leukemia. Blood Cancer J..

[11] Fianchi L., Leone G., Posteraro B., Sanguinetti M., Guidi F., Valentini C.G., Voso M.T., Pagano L. (2011). Impaired bactericidal and fungicidal activities of neutrophils in patients with myelodysplastic syndrome. Leuk. Res..

[12] Hueso T., Ekpe K., Mayeur C., Gatse A., Curt M.J.-C., Gricourt G., Rodriguez C., Burdet C., Ulmann G., Neut C. (2020). Impact and consequences of intensive chemotherapy on intestinal barrier and microbiota in acute myeloid leukemia: The role of mucosal strengthening. Gut Microbes.

[13] Stincardini C., Pariano M., D’Onofrio F., Fianchi L., Giordano A., Bellet M.M., Costantini C., Pagano L., Romani L., Renga G. Reducing Collateral Toxicity of Standard Therapy in Acute Myeloid Leukemias by Preserving Epithelial Barrier Function. Abstracts from 2022 Società Italiana di Patologia e Medicina Traslazionale (SIPMeT) Congress, Pathophysiology of Disease-September, 22nd–24th, 2022-Ancona..

[14] Fianchi L., Criscuolo M., Lunghi M., Gaidano G., Breccia M., Levis A., Finelli C., Santini V., Musto P., Oliva E.N. (2012). Outcome of therapy-related myeloid neoplasms treated with azacitidine. Leuk. Res..

[15] Pagano L., Caira M., Picardi M., Candoni A., Melillo L., Fianchi L., Offidani M., Nosari A. (2007). Invasive Aspergillosis in Patients with Acute Leukemia: Update on Morbidity and Mortality--SEIFEM-C Report. Clin. Infect. Dis..

[16] Caira M., Candoni A., Verga L., Busca A., Delia M., Nosari A., Caramatti C., Castagnola C., Cattaneo C., Fanci R. (2015). Pre-chemotherapy risk factors for invasive fungal diseases: Prospective analysis of 1,192 patients with newly diagnosed acute myeloid leukemia (SEIFEM 2010-a multicenter study). Haematologica.

[17] Cattaneo C., Marchesi F., Terrenato I., Bonuomo V., Fracchiolla N.S., Delia M., Criscuolo M., Candoni A., Prezioso L., Facchinelli D. (2022). High Incidence of Invasive Fungal Diseases in Patients with FLT3-Mutated AML Treated with Midostaurin: Results of a Multicenter Observational SEIFEM Study. J. Fungi.

[18] Pagano L., Caira M., Candoni A., Offidani M., Martino B., Specchia G., Pastore D., Stanzani M., Cattaneo C., Fanci R. (2010). Invasive aspergillosis in patients with acute myeloid leukemia: A SEIFEM-2008 registry study. Haematologica.

[19] Candoni A., Farina F., Perruccio K., Di Blasi R., Criscuolo M., Cattaneo C., Delia M., Zannier M.E., Dragonetti G., Fanci R. (2020). Impact of invasive aspergillosis occurring during first induction therapy on outcome of acute myeloid leukaemia (SEIFEM-12B study). Mycoses.

[20] Matthews A.H., Perl A.E., Luger S.M., Loren A.W., Gill S.I., Porter D.L., Babushok D.V., Maillard I.P., Carroll M.P., Frey N.V. (2022). Real-world effectiveness of CPX-351 vs. venetoclax and azacitidine in acute myeloid leukemia. Blood Adv..

